# Socio-Cultural Experiences of Exclusive Breastfeeding Among Banjar Mothers in Indonesia

**DOI:** 10.3390/healthcare14142110

**Published:** 2026-07-14

**Authors:** Dina Aprilia, Afra Hafny Noer, Retno Hanggarani Ninin, Zahrotur Rusyda Hinduan

**Affiliations:** 1 Faculty of Psychology, Universitas Padjadjaran Bandung, Jatinangor 45363, Indonesia; afra@unpad.ac.id (A.H.N.); rhninin@unpad.ac.id (R.H.N.); z.r.hinduan@unpad.ac.id (Z.R.H.); 2 Faculty of Ushuluddin and Humanities, Universitas Islam Negeri Antasari Banjarmasin, Banjarmasin 70235, Indonesia

**Keywords:** exclusive breastfeeding, cultural context, postpartum stress, social support

## Abstract

**Background**: Exclusive breastfeeding is recognized as an effective public health intervention; however, its practice is shaped by complex social, emotional, and cultural dynamics, particularly within collectivist societies. This study aims to explore in depth the meaning of exclusive breastfeeding in the early postpartum period within the socio-cultural context of the Banjar community with a focus on postpartum stress and the dynamics of social support. **Methods**: This qualitative study, grounded in a constructivist paradigm and designed as a case study, was conducted among 15 Banjar mothers aged 18–35 years with infants aged 0–6 weeks in Banjarmasin City and Banjar Regency, South Kalimantan, Indonesia. Data were collected through in-depth interviews and analyzed using thematic analysis following Braun and Clarke between May and August 2024. All interview data were translated and verified through collaborative review among bilingual researchers to ensure accuracy. **Results**: The analysis identified five main themes: breastfeeding knowledge, social support, postpartum stress, attitudes toward breastfeeding, and exclusive breastfeeding practices. Although some participants experienced formula introduction, breastfeeding practices were often described as efforts to maintain exclusive breastfeeding rather than continuous exclusive adherence. The findings reveal that breastfeeding is interpreted not only as nutritional provision but also as a relational, moral, and religious practice embedded within collectivist values and Islamic teachings. Social support is experienced as ambivalent, functioning as both a protective and normative force within extended family structures. Postpartum stress specifically related to breastfeeding emerges from limited early initiation of breastfeeding, surrounding formula-feeding dilemmas, and internal conflicts between feelings of guilt and commitment to breastfeeding. Maternal attitudes are shaped by personal commitment, religious beliefs, and critical reflections on medical interventions, while exclusive breastfeeding practices are enacted through adaptive strategies, breast milk management, and reinforcement from the social environment. **Conclusions**: This study provides a context-sensitive understanding of early postpartum exclusive breastfeeding as a culturally embedded maternal experience. By highlighting how social support, cultural expectations, postpartum stress, and maternal attitudes intersect to shape breastfeeding practices, the study contributes new insights for culturally grounded maternal health interventions in collectivist Muslim societies.

## 1. Introduction

Exclusive breastfeeding is widely recognized as one of the most effective public health interventions for improving infant survival and maternal health [1]. Despite global recommendations advocating exclusive breastfeeding during the early postpartum period, followed by continued breastfeeding up to two years or beyond [2,3], its practice remains suboptimal worldwide. Globally, only approximately 44% of infants receive exclusive breastfeeding in the early postpartum period [4], while in Indonesia, coverage is around 52.5% with substantial regional and cultural variation [3]. Although breastfeeding provides well-established benefits for infants and mothers, including reduced risks of postpartum hemorrhage, breast and ovarian cancer, and metabolic disorders [5], the gap between recommendations and actual practice highlights the need to understand breastfeeding beyond a purely knowledge-driven health behavior [4,6,7,8].

The postpartum period represents a critical transitional phase characterized by profound physiological, hormonal, and psychological changes in women’s lives [9,10]. During this period, mothers undergo the formation of a new maternal identity, often accompanied by physical exhaustion, sleep deprivation, and changes in interpersonal relationships [9,11]. These conditions may contribute to postpartum stress, anxiety, and mild-to-moderate depressive symptoms, all of which can negatively affect maternal emotional well-being [11,12]. Previous studies have demonstrated that postpartum stress is associated with reduced breastfeeding self-efficacy, perceived insufficient milk supply, and an increased likelihood of early formula feeding [13,14]. In addition, mothers may experience dilemmas between maintaining exclusive breastfeeding and introducing formula feeding due to perceived insufficient milk supply or concern about infant growth. However, existing research has largely approached postpartum stress as an individual psychological factor, with limited attention on how such stress is socially constructed and negotiated within broader relational and cultural contexts. Consequently, breastfeeding should be understood not merely as a biological or individual practice, but as a dynamic arena of psychological negotiation shaped by expectations, capabilities, and situational pressures [15].

Social support plays a critical role in facilitating breastfeeding [16]. Support from partners, family members, healthcare providers, and communities helps mothers cope with breastfeeding challenges and mitigate stress [17,18,19]. Its forms—emotional, informational, instrumental, and appraisal—can promote breastfeeding continuity. Nevertheless, the meaning and impact of social support are culturally mediated, particularly in collectivist societies where family expectations and hierarchical structures may create both support and pressure [20].

Existing studies predominantly focus on Western, individualistic contexts [8,21,22,23,24,25], emphasizing maternal autonomy, partner support, and healthcare guidance. These perspectives inadequately explain breastfeeding in collectivist societies, where decisions involve extended families, cultural norms, and communal expectations [26,27,28,29]. In Muslim-majority and collectivist settings, breastfeeding also carries moral and religious significance [30,31,32], and failure to meet these expectations may generate guilt and stress [33]. Furthermore, limited attention has been given to how breastfeeding-related dilemmas, particularly those involving formula-feeding decisions, are negotiated within socio-cultural and religious contexts.

In Indonesia, particularly among the Banjar ethnic community in South Kalimantan, extended family structures and hierarchical kinship systems strongly influence infant feeding decisions [27,34,35,36]. Exclusive breastfeeding is thus a culturally and religiously embedded practice shaped by family expectations and Islamic teachings [37,38]. Despite quantitative studies documenting knowledge, stress, and social support in Indonesia [39], qualitative research exploring mothers’ lived experiences remains limited.

Addressing this gap, this study aims to explore the early postpartum experiences, meanings, and social negotiations surrounding exclusive breastfeeding among Banjar mothers within their socio-cultural context, focusing on postpartum stress related to breastfeeding and the dynamics of social support.

## 2. Materials and Methods

### 2.1. Study Setting

Indonesia represents a socio-cultural context characterized by high diversity in ethnicity, culture, and religion, with a predominantly collectivist social structure. In such contexts, individual behavior, including maternal roles, is strongly influenced by social norms, family expectations, and community values. This study was conducted in South Kalimantan Province, focusing on breastfeeding mothers from the Banjar ethnic group, where cultural and religious influences are deeply embedded in everyday life. The region provides a relevant setting to examine how exclusive breastfeeding is shaped not only by individual factors but also by socio-cultural and relational dynamics within early postpartum experiences. To contextualize the study setting more systematically, the key socio-cultural characteristics relevant to breastfeeding practices are summarized in Table 1.

This study employs a qualitative approach grounded in a constructivist paradigm to explore how mothers construct and negotiate the meaning of exclusive breastfeeding within their socio-cultural context [42]. From this perspective, reality is understood as socially constructed through interactions between individuals and their environment, while knowledge emerges from subjective interpretations of lived experiences [43]. By framing the Banjar mothers’ early postpartum breastfeeding experiences as a bounded case, the study justifies the use of a qualitative case study design. This approach allows for systematic exploration of key themes related to breastfeeding knowledge, postpartum stress, social support, and maternal attitudes within a specific cultural and social context, while providing a nuanced and contextually grounded understanding of exclusive breastfeeding as a culturally embedded maternal practice.

### 2.2. Participants

This study was conducted in Banjarmasin City and Banjar Regency, South Kalimantan, Indonesia, which are recognized as core areas of Banjar culture with a predominantly Banjar ethnic population and accessible maternal and child healthcare services. The study focused on exploring exclusive breastfeeding as a socio-culturally embedded maternal experience among Banjar mothers during the early postpartum period.

Participant recruitment was conducted purposively to ensure that participants were relevant to the research objectives and could provide rich, contextually grounded information on breastfeeding experiences within the Banjar socio-cultural setting. Recruitment was carried out in collaboration with primary healthcare centers (Puskesmas) and community health posts (Posyandu). Healthcare personnel played a key role by informing the researcher when postpartum mothers meeting preliminary inclusion criteria visited the facilities, without disclosing any personal identifying information. This approach ensured the privacy and confidentiality of potential participants during the recruitment stage. Following identification, the researcher directly approached eligible mothers and their families to explain the study’s purpose, procedures, and ethical considerations. Eligibility was assessed according to the predefined inclusion and exclusion criteria (see Table 2), which targeted mothers of Banjar ethnicity aged 18–35 years with infants 0–6 weeks old, who were currently breastfeeding or intending to do so, and willing to provide informed consent. Mothers who agreed to participate provided written informed consent before any data collection occurred.

Initial meetings at the healthcare facilities were primarily used to establish rapport and arrange mutually convenient times and locations for interviews. In-depth interviews were subsequently conducted at locations chosen by participants, most commonly their homes, to ensure privacy, comfort, and confidentiality. During all interactions, care was taken to protect participants’ identities and maintain ethical standards, including anonymizing all data using participant codes, removing identifying information from transcripts, and securely storing digital and printed materials.

The inclusion and exclusion criteria were designed to capture early postpartum breastfeeding experiences among Banjar mothers in relatively stable health condition, thereby enabling a deeper understanding of the interaction between breastfeeding practices, cultural expectations, religious values, family dynamics, and maternal adaptation during the postpartum period. A total of 15 mothers participated in the study. The participant selection criteria are presented in Table 2, while detailed participant characteristics are summarized in Table 3. As presented in Table 3, participants demonstrated diverse socio-demographic and obstetric characteristics. Several participants had undergone cesarean delivery and had infants with low birth weight, while others experienced vaginal delivery with normal-birth-weight infants. Most participants lived in extended family settings, reflecting the collectivist culture of Banjar community. This diversity of characteristics provides a rich context for exploring variations in breastfeeding experiences.

### 2.3. Ethical Considerations

This study received ethical approval from the Research Ethics Committee of Universitas Padjadjaran, Indonesia (Approval Number: 474/UN6.KEP/EC/2024; approved on 29 April 2024). The study was conducted in accordance with the ethical principles outlined in the Declaration of Helsinki. Research permission was also obtained from the Banjarmasin City Health Office, which subsequently provided recommendations to several primary healthcare centers (Puskesmas) serving as recruitment sites for this study. Prior to participation, all participants received detailed explanations regarding the study objectives, procedures, potential risks and benefits, confidentiality measures, and their rights as research participants. Written informed consent was obtained from all participants before data collection commenced. Participation was entirely voluntary, and participants were informed of their right to withdraw from the study at any stage without any consequences. To ensure confidentiality and data protection, healthcare providers at the Puskesmas did not disclose participants’ personal identities to the researcher during the recruitment process. All interview recordings and transcripts were anonymized using participant codes, and any identifying information was removed from the dataset. Digital data were stored in password-protected files accessible only to the research team, while printed materials were securely stored in locked files to maintain participant privacy and data security.

### 2.4. Data Collection

Data were collected through semi-structured in-depth interviews using open-ended questions to explore participants’ lived breastfeeding experiences during the early postpartum period. A total of 15 primary interviews were conducted with mothers, and additional interviews were conducted separately with the husbands of 10 participants to capture the family perspective on breastfeeding support. All participants, including husbands, provided separate written informed consent prior to data collection. The interview guide was developed systematically through several stages, including defining the research objectives, identifying key themes and subthemes, constructing question frameworks, and refining interview items through expert review to ensure clarity, relevance, and cultural appropriateness. The interview was developed through a systematic and iterative process to ensure both conceptual rigor and contextual relevance. Building upon the initial identification of research objectives and key thematic areas, the preliminary interview framework was first discussed with academic supervisors to ensure alignment with the theoretical foundation, research design, and overall study objectives. This stage enabled the refinement of key constructs and clarification of the scope of inquiry.

Subsequently, an expert consultation was conducted through a focused group discussion (FGD) involving five experts in psychology, maternal and child health, and breastfeeding counseling. This process aimed to further evaluate the clarity, cultural appropriateness, and contextual relevance of the interview questions. Insights obtained from the FGD informed the refinement of key themes, the restructuring of question sequences, and the development of more effective probing strategies. Following these stages, a pilot interview was conducted with participants who shared similar characteristics with the target population to assess the comprehensibility, flow, and contextual suitability of the interview guide in real-life conditions. Feedback from the pilot interview was incorporated to make final adjustments prior to the main data collection phase.

The interviews focused on three main areas: (1) knowledge and understanding of breastfeeding and exclusive breastfeeding, (2) mothers’ subjective experiences of receiving or lacking social support, and (3) emotional dynamics during the postpartum period related to breastfeeding practices. Interviews began with broad guiding questions and were followed by probing techniques to elicit deeper explanations and detailed narratives.

Interviews were conducted at locations mutually agreed upon with participants, most commonly in participants’ homes to ensure comfort and privacy, and lasted approximately 45–90 min. Repeat interviews were conducted with three participants to clarify or expand on emerging themes. All interviews were audio-recorded with participants’ consent. All participant data were anonymized prior to analysis, and all personal identifiers were removed to ensure confidentiality and data protection. In addition, limited participant observation was conducted to capture interactions between mothers, infants, and family members, accompanied by field notes documenting relevant socio-cultural contexts. The transcription process was assisted by five research assistants with undergraduate backgrounds in psychology who were native speakers of the Banjar language. This facilitated a more accurate understanding of local expressions and culturally embedded meanings. Interviews conducted in the Banjar language were translated into Indonesian and verified through collaborative discussions between the principal researcher and bilingual research assistants to ensure linguistic accuracy and preservation of meaning. All transcripts and translated materials were repeatedly reviewed by the principal researcher before analysis to ensure data quality and consistency [44]. To enhance data credibility, source triangulation was applied through additional interviews with participants’ husbands as a form of cross-validation within the family context [45]. This approach strengthened the trustworthiness and contextual interpretation of the findings.

### 2.5. Data Analysis

Data were analyzed using thematic analysis following the six-phase framework proposed by Braun and Clarke [43]. All interview and observational transcripts were reviewed repeatedly to achieve familiarity with the data, and initial open coding was conducted by the principal researcher using NVivo software version 12 (QSR International/Lumivero) for efficient data management and retrieval.

**Figure 1:**
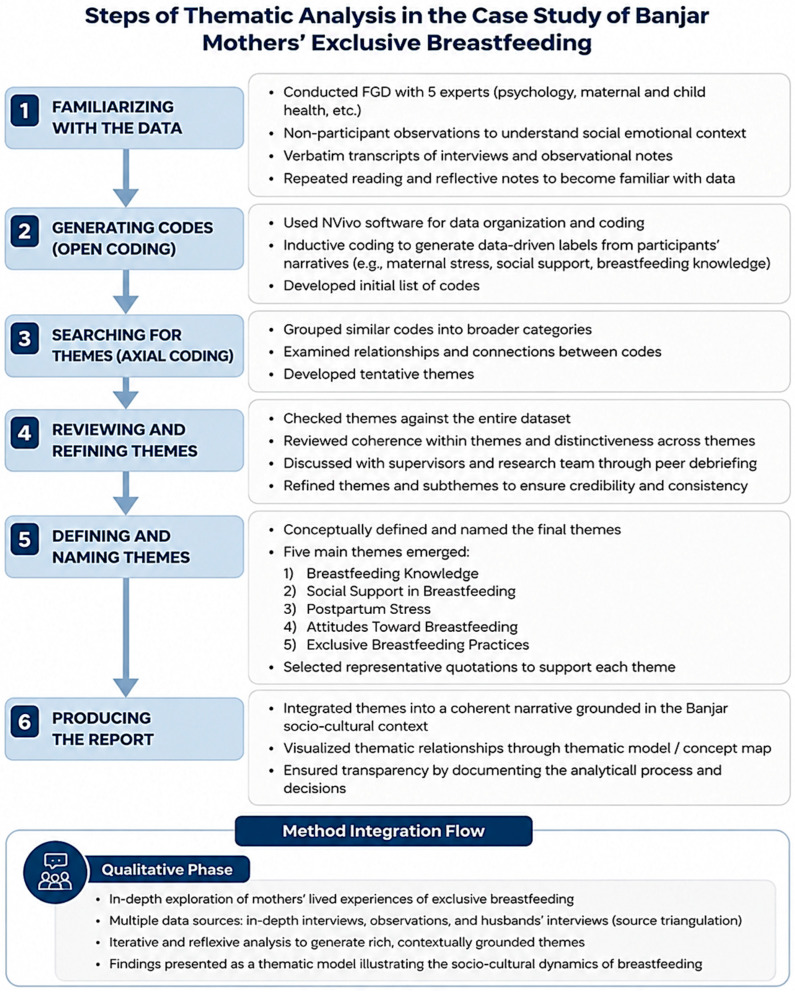
Thematic analysis process.

Figure 1 illustrates the application of Braun and Clarke’s (2006) [43] six-phase framework of thematic analysis used in this study. The process began with familiarization with the data through repeated reading of interview transcripts, field notes, non-participant observations, and FGD outputs with experts in maternal and child health. This was followed by generation of initial codes using NVivo software through an inductive, data-driven approach. Codes were then organized into broader categories during axial coding to identify relationships and patterns across participants’ narratives. In the fourth phase, themes were reviewed and refined through iterative comparison with the full dataset, peer debriefing, and supervisory discussions to ensure coherence and consistency. The fifth phase involved defining and naming final themes, resulting in five overarching themes: breastfeeding knowledge, social support in breastfeeding, postpartum stress, attitudes toward breastfeeding, and exclusive breastfeeding practices. Finally, the analysis was synthesized into a coherent narrative and conceptual representation of exclusive breastfeeding within the socio-cultural context of Banjar mothers, supported by methodological documentation to ensure transparency and trustworthiness.

Codes were iteratively grouped into categories and abstracted into broader themes through constant comparison across participants and observational data. An initial codebook was developed and refined throughout the analysis to maintain conceptual clarity and consistency. Peer debriefing and regular discussions with supervisors and colleagues with qualitative research expertise ensured transparency, consistency, and confirmability of coding decisions. Discrepancies in interpretation were resolved through consensus. Thematic relationships were visualized using conceptual mapping, and representative participant quotations were used to support interpretive transparency. Data adequacy was assessed using the principle of information power, considering the specificity of the participant group, the focused study aim, the quality of dialogue, and the depth of narratives. Analysis continued until sufficient depth and variation in experiences relevant to the research objectives were achieved. This streamlined approach aligns with COREQ and SRQR standards, emphasizing transparency, methodological rigor, and contextually grounded interpretation (Figure 1).

### 2.6. Trustworthiness

To ensure the trustworthiness of the findings, this study applied the criteria proposed by Lincoln and Guba [44] including credibility, transferability, dependability, and confirmability. Data source triangulation was employed by combining three sources of data: (1) in-depth semi-structured interviews with 15 mothers, (2) limited participant observations of mother–infant and family interactions conducted over a total of 20 h across all participants, and (3) supplementary interviews with 10 husbands of participating mothers. Importantly, the mothers’ interviews constituted the core dataset for coding and theme development, while husband interviews and observational data were treated as contextual and triangulation datasets rather than independent analytic units. These triangulation data were used to support and contextualize the mothers’ narratives rather than being analyzed as independent datasets. Specifically, husband interviews were analyzed to corroborate, contrast, and enrich mothers’ narratives regarding breastfeeding support, household decision-making, and family expectations. These data were not coded into separate standalone themes but were integrated during the interpretation stage to refine existing categories derived from mothers’ accounts. Similarly, participant observation data were used to contextualize and validate behavioral and relational dynamics observed in breastfeeding practices within natural settings. Field notes were incorporated into the analytical process to support interpretation of emerging codes and to enhance understanding of socio-cultural interactions that were not always explicitly articulated in interviews. Both husband interviews and observational data were therefore used to strengthen interpretive depth, clarify contextual meaning, and enhance triangulation, rather than to generate independent thematic structures. All datasets were managed using NVivo software and systematically cross-referenced during analysis. Observational notes and husband interview insights were compared with mothers’ narratives to triangulate evidence, enrich interpretation, and enhance thematic depth. This ensured that the final themes primarily reflected mothers’ lived experiences while being contextually validated through multiple data sources.

Member checking was conducted by providing participants with a summary of their own interview findings and preliminary thematic interpretations. Participants were invited to comment on, clarify, or elaborate on the summaries, ensuring that the interpretations accurately reflected their experiences. Feedback obtained during member checking was incorporated into the analysis, enhancing the credibility and authenticity of the findings. Peer debriefing involved regular discussions with qualitative research experts and academic supervisors to critically review the analytical process, coding decisions, and theme development. This iterative dialogue provided an external check on interpretation and minimized the influence of researcher bias. Transferability was supported through thick descriptions of the research setting, participant characteristics, and the socio-cultural context of the Banjar community, enabling readers to assess the applicability of findings to other contexts.

Dependability was maintained through a systematic audit trail documenting all stages of the research process, including data collection procedures, coding development, codebook revisions, analytical decisions, and theme refinement. Confirmability was enhanced through researcher reflexivity. The principal researcher, a psychologist with prior experience in maternal and child health research, acknowledged her professional background, personal beliefs, and insider/outsider positionality in relation to the Banjar community. While being partially an insider in terms of cultural familiarity, the researcher recognized potential biases stemming from her professional and academic perspectives. Reflexive practices included maintaining a reflexive journal, documenting analytic decisions, and critically examining personal assumptions that could influence interpretation. Regular reflective discussions with research peers were conducted to identify potential biases, challenge interpretations, and ensure transparency in data analysis. The research team consciously considered how their positionality—such as familiarity with participants’ language, norms, and healthcare contexts—might shape interactions, probing, and interpretation, thereby reinforcing the confirmability and rigor of the study. By combining triangulation, data integration, member checking, peer debriefing, thick description, audit trails, and reflexive practices, this study ensured that findings were credible, contextually grounded, and transparently derived from participants’ lived experiences while minimizing the influence of researcher bias.

## 3. Results

### 3.1. Coding and Theme Development Process

The coding and theme development process was conducted iteratively using an inductive thematic analysis approach, allowing themes to emerge directly from participants’ narratives rather than being imposed a priori. Initial open coding was performed line-by-line on the interview transcripts, generating data-driven labels that reflected mothers’ lived experiences regarding breastfeeding knowledge, social support, postpartum stress, attitudes, and breastfeeding practices. During this process, coding was conducted without preconceptions, enabling new patterns, insights, and nuanced experiences to surface naturally from the data.

Following initial coding, codes were grouped into categories and subcategories through constant comparison and iterative refinement. These categories were then abstracted into broader themes based on recurring patterns across participants, with the final thematic structure reflecting the complexity of exclusive breastfeeding experiences among Banjar mothers. Throughout the process, transparency and reflexivity were emphasized: analytic decisions, code definitions, and theme development were documented, and peer discussions with academic supervisors and the research team ensured rigor while preserving inductive reasoning.

The resulting analysis yielded five main themes, each representing a distinct but interconnected dimension of exclusive breastfeeding within the socio-cultural context of Banjar mothers. Table 4 summarizes the main themes, key meanings, and representative participant quotations.

The thematic analysis findings reveal that exclusive breastfeeding among Banjar mothers is not merely a health-related behavior but a multidimensional experience shaped by cognitive, emotional, and socio-cultural factors. Breastfeeding knowledge emerges as a foundational element that informs maternal understanding, yet it is deeply intertwined with personal experiences, perceived challenges, and adaptive strategies. The presence of both physical and psychological barriers highlights that knowledge alone is insufficient to sustain breastfeeding practices, as mothers must continuously negotiate their capabilities and limitations within real-life conditions. Furthermore, the identification of structured planning, such as intended breastfeeding duration and complementary feeding, indicates that mothers actively engage in forward-looking decision-making processes. Social support and postpartum stress appear as interconnected forces influencing maternal attitudes and behaviors. While support from family members and healthcare providers enhances confidence and continuity in breastfeeding, it simultaneously carries normative expectations that may contribute to emotional pressure. Postpartum stress, particularly arising from medical conditions and feeding dilemmas, reflects an internal conflict between ideal breastfeeding practices and situational constraints. These dynamics shape maternal attitudes, which are characterized by a combination of personal commitment, religious beliefs, and critical reflection on medical advice. Ultimately, exclusive breastfeeding practices are enacted through adaptive strategies and reinforced by supportive environments, suggesting that breastfeeding behavior is a socially negotiated and context-dependent process rather than a purely individual decision.

### 3.2. Analysis and Interpretation of Themes

i. Theme 1: Breastfeeding Knowledge

The findings indicate that breastfeeding knowledge among Banjar mothers is not limited to factual understanding but represents a dynamic and experiential construct shaped by cognitive, emotional, and contextual factors. Mothers demonstrate a foundational awareness of exclusive breastfeeding, particularly regarding its definition and duration, which is primarily acquired through healthcare providers. As expressed by one participant:

“I know that exclusive breastfeeding means giving only breast milk for early postpartum period, without adding formula or other foods” (Mother 3). However, this knowledge is continuously reinterpreted through personal experiences during the early postpartum period. Breastfeeding is perceived not only as a biological function but also as an emotional and relational process, as reflected in the following statement:

“It is a process that must be taken slowly because at first it feels painful and exhausting, but over time it creates a closer bond with the baby” (Mother 1).

**Figure 2:**
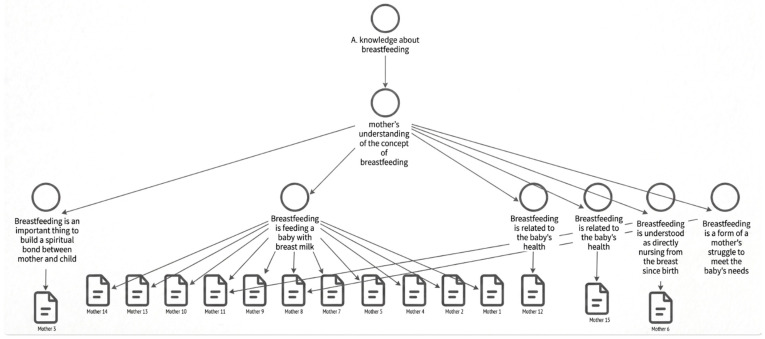
Mother’s understanding of the concept of breastfeeding.

In line with this, Figure 2 illustrates Banjar mothers’ understanding of breastfeeding as a socially and culturally constructed concept shaped by both formal knowledge and lived experience. At the core, “knowledge about breastfeeding” informs a central understanding that breastfeeding is not only feeding a baby with breast milk, but also an essential practice for promoting child health, a form of direct nursing from birth, and an important means of building a spiritual and emotional bond between mother and child. In addition, breastfeeding is also perceived as a maternal responsibility and as a challenging process of meeting the baby’s needs. Overall, this conceptualization reinforces the multidimensional nature of breastfeeding knowledge, integrating biomedical understanding with emotional meaning, relational bonding, and socio-cultural–religious values.

Furthermore, the presence of both physical and psychological barriers reveals that knowledge alone does not guarantee successful breastfeeding practices. Mothers actively negotiate their understanding in response to challenges such as pain, fatigue, and perceived insufficient milk supply. One participant noted, “At the beginning, the milk did not come out much, the baby cried often, and I became stressed” (Mother 2), while another expressed concern: “I often worry that my milk is not enough and that my baby might be hungry” (Mother 4). These experiences illustrate how knowledge is shaped through ongoing interaction with real-life constraints. In response, mothers adopt adaptive strategies, including seeking professional guidance and maintaining breastfeeding efforts, as indicated by a participant who stated, “I often consult with the midwife and ask questions when feeling uncertain” (Mother 6). In addition, forward-looking considerations, such as planned breastfeeding duration and complementary feeding, demonstrate that mothers engage in anticipatory decision-making, for example, “I want to breastfeed for two years, but I will also consider my condition later” (Mother 7). These findings suggest that breastfeeding knowledge operates as a flexible and context-dependent framework that interacts with emotional experiences and practical realities rather than as a purely informational construct.

ii. Forms and Sources of Social Support

The findings indicate that breastfeeding mothers receive support from multiple sources, both within and beyond the family environment. Among these, husbands emerge as the most prominent source of support, particularly in providing emotional reassurance and practical assistance during the breastfeeding process. Extended family members, especially mothers and mothers-in-law, also play a crucial role by sharing knowledge and prior experiences related to breastfeeding. In addition, healthcare professionals, such as midwives and nurses, contribute through guidance and education. This multidimensional support network reflects the collectivist nature of the Banjar community, where maternal experiences are embedded within broader relational structures. As one participant explained, “I received a lot of help from my husband and my mother, especially at the beginning when I was still confused about breastfeeding” (Mother 5). Another participant emphasized the importance of spousal support, stating, “My husband helps take care of the baby when I need to express breast milk, so I feel less overwhelmed” (Mother 3).

**Figure 3:**
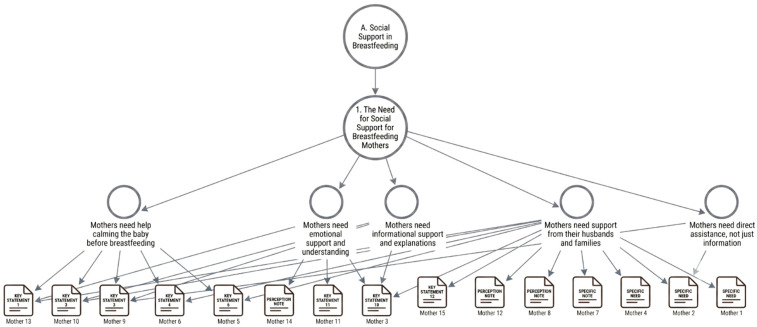
The need for social support for breastfeeding mothers.

In line with these findings, Figure 3 presents a conceptual map of social support in breastfeeding among Banjar mothers, highlighting “the need for social support for breastfeeding mothers” as the central theme under the broader category of social support in breastfeeding. The diagram shows that mothers require multiple interconnected forms of support, including help in calming the baby before breastfeeding, emotional support and understanding, and informational support and clear explanations regarding breastfeeding practices, as well as both direct practical assistance and complementary guidance. It further emphasizes that husbands and family members are key sources of support, playing an important role in providing both emotional reassurance and hands-on assistance. Overall, this conceptualization illustrates that social support is multidimensional and relational, combining emotional, informational, and instrumental dimensions that collectively facilitate successful breastfeeding practices.

The forms of support provided to mothers are diverse and encompass emotional, informational, and instrumental dimensions. Emotional support is reflected in encouragement, reassurance, and motivational reinforcement, which helps mothers maintain confidence in their ability to breastfeed. Informational support is delivered through advice regarding breastfeeding techniques, positioning, feeding frequency, and ways to manage breastfeeding difficulties. Instrumental support includes tangible assistance, such as helping with household tasks or caring for the infant, allowing mothers to rest and recover. These forms of support operate synergistically in reducing maternal burden and facilitating breastfeeding continuity. One participant noted, “My husband helps with household chores, so I can focus on breastfeeding and feel more relaxed” (Mother 1), while another highlighted professional guidance, stating, “The midwife taught me the correct breastfeeding position, which made me more confident” (Mother 4).

Importantly, the findings suggest that social support functions not only as a practical resource but also as a relational mechanism that shapes maternal confidence and emotional stability. The presence of supportive individuals enhances mothers’ sense of security and strengthens their commitment to breastfeeding, whereas limited or inconsistent support may lead to uncertainty and emotional strain. As one participant reflected, “When I feel supported, I become more confident and motivated to continue breastfeeding” (Mother 1). In contrast, another participant expressed concern about insufficient support, noting that “without support, I would feel overwhelmed and unsure whether I could continue breastfeeding” (Mother 2). These findings highlight that social support is a context-dependent and relationally constructed phenomenon that significantly influences how mothers interpret challenges and sustain breastfeeding practices within collectivist cultural settings.

iii. Theme 3: Postpartum Stress

The findings reveal that postpartum stress among Banjar mothers emerges as a complex emotional response shaped by physical conditions, healthcare practices, and concerns about infant well-being. Cesarean delivery appears to be a significant trigger of emotional distress, particularly due to physical limitations and restricted opportunities for early breastfeeding initiation. The absence or suboptimal implementation of early initiation of breastfeeding (IMD) contributes to feelings of frustration and emotional discomfort. One participant described the situation as follows: “The healthcare facility where I gave birth did not have a standard procedure for early breastfeeding initiation… even though it was possible, it was not ideal, maybe only 10–15 min” (Mother 4). In addition, separation from the infant after delivery further intensified emotional strain, as reflected in the following statement: “I felt upset and sad, but I could not force the healthcare staff… that night I felt restless, even though I could still sleep” (Mother 1). These experiences indicate that institutional and clinical conditions play a crucial role in shaping early postpartum emotional responses.

**Figure 4:**
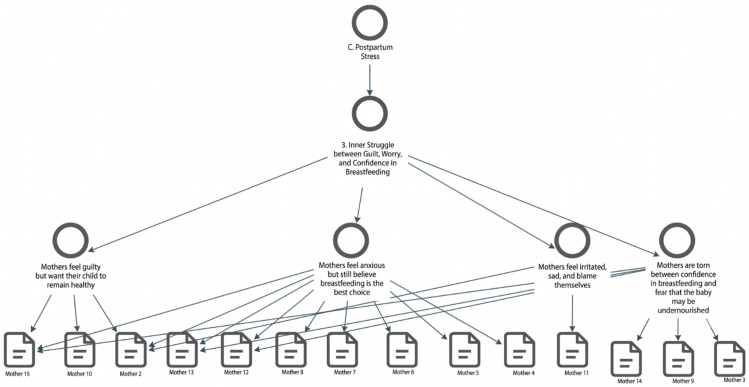
The inner struggle between guilt, worry, and confidence in breastfeeding.

In line with these findings, Figure 4 illustrates postpartum stress among Banjar mothers as a multidimensional and emotionally complex experience centered on an “inner struggle between guilt, worry, and confidence in breastfeeding.” The diagram shows that postpartum stress originates from broader postpartum conditions and is expressed through several interconnected emotional states, including mothers feeling guilty yet wanting their child to remain healthy, feeling anxious while still believing breastfeeding is the best choice, feeling irritated or sad, and blaming themselves, as well as being torn between confidence in breastfeeding and fear that the baby may be undernourished. Overall, this conceptualization highlights that postpartum stress is not a single emotional reaction, but a dynamic psychological tension shaped by competing emotions, maternal responsibility, and concerns for infant well-being within the breastfeeding experience.

Postpartum stress is further intensified by psychological dilemmas related to feeding decisions, particularly when mothers are advised to use infant formula due to perceived insufficient breast milk or concerns about infant growth. This situation generates internal conflict between maintaining exclusive breastfeeding and ensuring the infant’s health. As one participant expressed, “It was an intense inner struggle, I wondered whether my ego would prevent my child from growing properly” (Mother 7). The pressure is often reinforced by family members, as illustrated by another statement: “My husband also started to hesitate when told about possible effects on the baby’s brain development” (Mother 7). These conditions contribute to feelings of guilt, anxiety, and uncertainty, which are central to the postpartum experience. One mother shared, “I felt guilty…I was worried because we cannot turn back time” (Mother 10). However, despite these emotional challenges, some mothers demonstrated resilience and maintained their commitment to breastfeeding, particularly when supported by healthcare professionals. As noted by one participant, “I felt reassured by the doctor…even for low birth weight babies, formula was not necessary, I was encouraged to continue breastfeeding” (Mother 7). These findings highlight that postpartum stress is not a singular emotional state but a dynamic interplay between guilt, concern, and belief in breastfeeding, shaped by both personal and contextual factors.

iv. Theme 4: Attitudes toward Breastfeeding

The findings indicate that mothers’ attitudes toward breastfeeding are shaped by an integration of personal values, beliefs, and lived experiences, resulting in a multidimensional orientation that extends beyond cognitive understanding. Breastfeeding is perceived not merely as a nutritional practice but as a moral commitment and personal responsibility toward the infant. This perception is reinforced by strong beliefs regarding the irreplaceable value of breast milk, as reflected in one participant’s statement: “Breast milk is a substance that cannot be replaced by anything, especially colostrum… it is like liquid gold, no other food can match it” (Mother 14). Such beliefs motivate mothers to persist in breastfeeding despite experiencing fatigue and emotional strain. Another participant expressed the emotional significance of breastfeeding by stating, “Breastfeeding is exhausting, but when I see my baby feeding, it makes me happy, and as a mother, I feel fulfilled” (Mother 4). These findings suggest that breastfeeding is constructed as a meaningful emotional experience that reinforces maternal identity and commitment.

**Figure 5:**
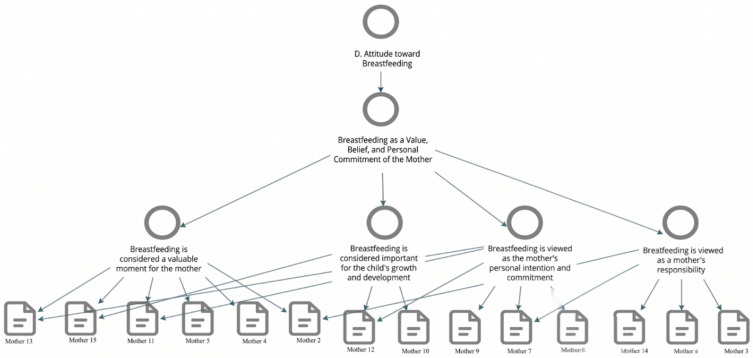
Breastfeeding as a value, belief, and personal commitment of mothers.

In line with these findings, Figure 5 presents Banjar mothers’ attitudes toward breastfeeding as a value-laden and personally meaningful construct shaped by belief systems and maternal commitment. At the core, breastfeeding is framed as “breastfeeding as a value, belief, and personal commitment of the mother,” which further branches into several interconnected meanings. Mothers view breastfeeding as a valuable moment in motherhood, as an important practice for the child’s growth and development, as an expression of personal intention and commitment, and as a fundamental maternal responsibility. Overall, this conceptualization demonstrates that attitudes toward breastfeeding are not merely cognitive evaluations, but deeply rooted in emotional meaning, moral obligation, and self-defined maternal roles within the socio-cultural context.

In addition to these internalized values, mothers also demonstrate a critical stance toward medical interventions that are not perceived as clearly justified, particularly regarding the recommendation of formula feeding. This critical attitude emerges from the tension between institutional practices and mothers’ intentions to maintain exclusive breastfeeding. One participant explained, “We were asked to buy infant formula to be given, but fortunately the doctor did not recommend it because I wanted to breastfeed” (Mother 1). Such experiences encourage mothers to assert greater control over feeding decisions, as illustrated by another statement: “I made it clear to the healthcare staff that if formula was to be given, they had to confirm it with us first” (Mother 4). At the same time, exclusive breastfeeding is interpreted as both a rational and emotional choice. Rational considerations include perceived health benefits and economic advantages, as reflected in the statement, “If we can breastfeed, we do not need to buy infant formula” (Mother 2). Emotionally, breastfeeding is associated with feelings of responsibility and anticipated regret, as one mother expressed, “It is the feeling of guilt that I worry about, because we cannot turn back time” (Mother 4). These findings highlight that maternal attitudes toward breastfeeding are formed through the interplay of belief systems, experiential learning, and emotional attachment, positioning breastfeeding as both a deliberate and deeply personal decision.

v. Theme 5: Exclusive Breastfeeding Practices

The findings demonstrate that exclusive breastfeeding practices among Banjar mothers are characterized by adaptive and context-sensitive behaviors, particularly in situations involving low-birth-weight infants. Mothers actively adjust their feeding strategies to accommodate the physical limitations of their infants, reflecting a high level of responsiveness and commitment to maintaining breastfeeding. When direct breastfeeding is not feasible, mothers employ alternative approaches such as expressing breast milk to ensure continued nutritional intake. As one participant explained, “The baby was very small and could not breastfeed for long, so I had to express my breast milk first” (Mother 2). In addition, mothers increase feeding frequency, even in small amounts, as part of a gradual and sustained effort to support infant growth. This is reflected in the statement, “Even if it is only a little, it is okay, as long as it is breast milk and given regularly” (Mother 2). These findings indicate that breastfeeding practices are not fixed behaviors but continuously adapted in response to situational challenges, demonstrating maternal agency in overcoming constraints.

**Figure 6:**
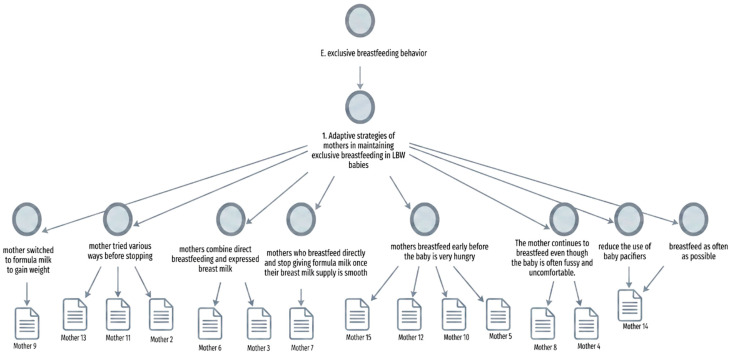
Mothers’ adaptive strategy in maintaining exclusive breastfeeding for low-birth-weight infants.

In line with these findings, Figure 6 presents adaptive strategies used by Banjar mothers to maintain exclusive breastfeeding, particularly in infants with low birth weight, as a dynamic and context-responsive behavioral process. At the core, “exclusive breastfeeding behavior” is supported by mothers’ adaptive strategies in sustaining breastfeeding, which are expressed through multiple practical approaches. These include switching temporarily to formula feeding to support infant weight gain, trying various methods before discontinuing certain practices, combining direct breastfeeding with expressed breast milk, temporarily introducing formula until milk supply stabilizes, breastfeeding early before the baby becomes very hungry, continuing breastfeeding even when the baby is fussy or uncomfortable, reducing or avoiding the use of pacifiers, and breastfeeding as frequently as possible. Overall, this conceptualization shows that exclusive breastfeeding is not a fixed practice, but an adaptive and flexible process shaped by maternal effort, infant condition, and ongoing adjustments to feeding challenges.

Beyond adaptive strategies, mothers engage in practical efforts to sustain exclusive breastfeeding, including skin-to-skin contact, breast milk management, and consultation with healthcare professionals. Skin-to-skin contact is perceived as a technique that not only strengthens emotional bonding but also stimulates milk production, as one participant noted, “I often do skin-to-skin contact so that the milk flows better and the baby feels calmer” (Mother 4). Mothers also perform routine milk expression to maintain supply despite fatigue, as illustrated by the statement, “Even though I am tired, I still pump breast milk so there is always enough supply for the baby” (Mother 3). Professional guidance further supports these practices, with one mother explaining, “I keep asking the midwife about the correct breastfeeding position so the baby does not have difficulty feeding” (Mother 4). Importantly, the social environment plays a reinforcing role in sustaining these behaviors. Emotional encouragement from family members, particularly husbands, strengthens maternal confidence, as reflected in the statement, “My husband keeps telling me to continue breastfeeding, so I feel stronger” (Mother 5). Similarly, validation from healthcare providers reinforces mothers’ commitment, as one participant stated, “The doctor told me to keep breastfeeding, that a small baby is not a reason to stop” (Mother 3). These findings suggest that exclusive breastfeeding practices are co-constructed through individual effort and social reinforcement, highlighting the interplay between maternal agency and supportive environments in sustaining breastfeeding behavior.

## 4. Discussion

### 4.1. Breastfeeding Knowledge Among Banjar Mothers Is Socially and Culturally Constructed in Interpreting Exclusive Breastfeeding Practices

This study found that Banjar mothers generally possess foundational knowledge of exclusive breastfeeding, particularly regarding the recommended practice of providing only breast milk during the early postpartum period. This knowledge is primarily acquired through interactions with healthcare professionals during antenatal and postnatal care, reinforcing the role of institutional health education in shaping maternal understanding [5,46,47]. However, knowledge depth varies, with some mothers demonstrating limited understanding of the physiological mechanisms and long-term developmental benefits of breastfeeding [20,23]. Importantly, these findings extend the existing breastfeeding literature by demonstrating that exclusive breastfeeding should not be understood solely as an individual health behavior, but rather as a socially negotiated and culturally embedded practice shaped by relational, emotional, and religious dimensions.

Furthermore, the findings highlight that breastfeeding-related dilemmas, particularly those involving formula-feeding decisions, represent a critical site of negotiation where mothers balance biomedical recommendations, infant health concerns, and socio-cultural expectations. A key finding is that breastfeeding knowledge extends beyond cognitive awareness and is embedded in relational and emotional meanings. Mothers perceive breastfeeding as an experiential process facilitating bonding with their infants, consistent with concepts of embodied knowledge and the lived maternal experience [7,8,24]. Knowledge is thus dynamically constructed through both formal instruction and early postpartum experiences, particularly for primiparous mothers who describe learning through trial, adaptation, and emotional adjustment [9,11,12].

Religious and cultural values, especially Islamic teachings recommending breastfeeding up to two years, add a moral and ethical dimension to breastfeeding, which can strengthen motivation but also generate psychological pressure when mothers face challenges [30,31,32]. In the Banjar context, strong kinship structures and collectivist norms further shape knowledge acquisition and breastfeeding practices [27,28,36,41,48].

These findings highlight that breastfeeding knowledge is socially negotiated, integrating formal health information, embodied experience, cultural norms, and relational influences. This underscores the importance of culturally sensitive breastfeeding interventions that consider both the social and emotional dimensions of maternal learning. Future research could examine strategies to enhance conceptual understanding, support maternal adaptation in the early postpartum period, and evaluate interventions that leverage family and community networks to reinforce exclusive breastfeeding practices.

### 4.2. Social Support Among Banjar Mothers Functions as a Multidimensional and Ambivalent Mechanism in Shaping Early Postpartum Breastfeeding Practices

This study found that social support plays a critical and multifaceted role in shaping early postpartum breastfeeding practices among Banjar mothers. Mothers receiving consistent support from husbands, extended family, and healthcare professionals demonstrated greater confidence and ability to maintain breastfeeding, whereas limited support was associated with fatigue, psychological distress, and increased risk of discontinuation [18,23,49]. These findings align with Dennis and Falah-Hassani [12], who describe social support as a buffer against postpartum stress, enhancing maternal resilience and well-being.

Husbands were identified as primary sources of emotional and instrumental support, consistent with Brown and Davies [15]. However, in the Banjar context, support networks are broader than the partner alone, including mothers, mothers-in-law, and healthcare providers, reflecting the collectivist structure of caregiving and decision-making [22,25,27,28,41]. This highlights that breastfeeding is a socially negotiated practice influenced by multiple actors rather than individual maternal autonomy.

An important contribution of this study lies in revealing the ambivalent nature of social support in collectivist settings. While extended family can provide reassurance and practical assistance, it may also impose normative pressures that conflict with medical guidance, creating tension in maternal decision-making [20,29,35]. These findings suggest that social support operates not only as a facilitating resource but also as a normative force that may impose expectations on mothers, particularly within collectivist family structures. This dual role underscores the need to consider not only the presence but also the quality and cultural alignment of support in breastfeeding interventions.

Mothers’ emotional responses further demonstrate that support influences psychological well-being, with supported mothers reporting higher confidence and motivation, while those lacking support experience anxiety and discouragement [11,12]. These findings suggest that breastfeeding interventions should adopt a family-centered approach, engaging husbands, extended family, and healthcare providers to strengthen culturally sensitive support networks and enhance early postpartum breastfeeding experiences [26,28].

### 4.3. Postpartum Stress Among Banjar Mothers Represents a Multidimensional Emotional Experience That Shapes Breastfeeding Meaning and Decision-Making

This study found that postpartum stress among Banjar mothers is a complex, multidimensional experience shaped by physical conditions, healthcare practices, and socio-cultural expectations during the early postpartum period. Key stressors included limitations in early breastfeeding initiation due to cesarean delivery, surrounding formula-feeding dilemmas, and internal conflicts between feelings of guilt and commitment to exclusive breastfeeding [11,12].

Cesarean delivery disrupted early initiation of breastfeeding (IMD), generating emotional distress linked to both physiological limitations and perceived deviations from ideal maternal practices. This aligns with Figueiredo et al. [50], who noted associations between postpartum stress, reduced breastfeeding self-efficacy, and concerns about milk sufficiency. In the Banjar context, where breastfeeding carries moral and religious significance, these challenges are interpreted as value-laden “failures,” consistent with Liamputtong’s [32] concept of moral distress and the collectivist expectation of responsible maternal behavior [27,28].

Psychological dilemmas around formula use further highlight stress as a negotiated process, balancing medical recommendations with cultural and personal commitments to exclusive breastfeeding [15,25,33]. This underscores that maternal identity and breastfeeding decisions are socially and morally embedded, rather than purely individual choices. Building on this, these findings further indicate that postpartum stress should not be understood solely as an individual psychological condition, but as relationally and contextually constructed experience shaped by healthcare practices, family dynamics, and cultural expectations. Importantly, postpartum stress did not necessarily lead to breastfeeding discontinuation. Its impact was mitigated by supportive healthcare interactions, particularly empathetic guidance from doctors and midwives aligned with evidence-based practices [12,14,23]. These findings emphasize that maternal emotional regulation is co-constructed through relational networks, including family and healthcare systems, which are central in collectivist societies such as the Banjar community.

These insights suggest that interventions to support breastfeeding should address emotional as well as technical needs, integrating culturally sensitive guidance, family involvement, and responsive healthcare support to enhance maternal confidence and resilience during the early postpartum period. Future research could further explore how structured support systems mediate postpartum stress across diverse cultural contexts.

### 4.4. Attitudes Toward Exclusive Breastfeeding Among Banjar Mothers Reflect a Complex Integration of Moral Values, Religious Beliefs, and Lived Early Postpartum Experiences

This study found that maternal attitudes toward breastfeeding are shaped by a combination of personal values, religious beliefs, and early postpartum experiences. Breastfeeding is perceived not merely as a lifestyle or health choice, but as a moral and religious commitment reflecting maternal identity and responsibility [30,31,32]. Within this context, breastfeeding is not merely a personal preference but is closely linked to moral responsibility and religious values, reinforcing maternal commitment beyond practical considerations. Attitudes are multidimensional, encompassing cognitive, affective, and conative components. Cognitively, mothers recognize the health benefits of breastfeeding for both infants and themselves [2,5]. Affectively, breastfeeding evokes positive emotions such as pride and bonding, reinforcing maternal identity. Conatively, mothers demonstrate persistent efforts to maintain breastfeeding despite challenges [8,15].

A notable finding is mothers’ critical evaluation of medical recommendations, particularly regarding formula feeding. Rather than passively accepting advice, mothers exercise agency and informed decision-making, reflecting active negotiation within a collectivist context [25,27,28]. This suggests that maternal autonomy can coexist with collectivist values, and that cultural context shapes how agency is exercised [29].

Emotionally, mothers frequently anticipate guilt or regret when unable to fulfill breastfeeding intentions, reflecting moral, social, and cultural pressures [35,36]. These emotional dimensions act as both motivation and psychological stress, emphasizing the need for interventions that address not only informational and practical support, but also emotional and moral aspects of breastfeeding within culturally specific contexts.

Interventions should support maternal autonomy, integrate family and community networks, and provide culturally sensitive guidance that considers both emotional and moral dimensions. Future research could examine how structured support programs can balance cultural expectations with evidence-based practices to enhance maternal confidence and early postpartum breastfeeding outcomes.

### 4.5. Exclusive Breastfeeding Practices Among Banjar Mothers Are Realized Through Dynamic Interactions Between Knowledge, Social Support, Postpartum Stress, and Maternal Attitudes

This study found that exclusive breastfeeding practices among Banjar mothers during the early postpartum period are dynamic and socially constructed, emerging from the interaction of knowledge, social support, postpartum stress, and maternal attitudes [49]. Practices were adaptive responses to evolving physiological, emotional, and social conditions, highlighting that breastfeeding behavior is context-dependent and relational rather than a linear health behavior.

Mothers of low-birth-weight infants employed flexible strategies, including expressing milk, increasing feeding frequency, and skin-to-skin contact, reflecting both practical problem-solving and strong moral and cultural commitment to breastfeeding [27,28]. These adaptive practices were reinforced by collectivist norms, emphasizing shared responsibility for infant care rather than individual maternal autonomy [27,29].

Breast milk management—routine expression and storage—demonstrated maternal competence, self-efficacy, and commitment, supporting findings by Fallon et al. [14] that maternal confidence predicts sustained breastfeeding under stress. Social influences, including encouragement from husbands, assistance from extended family, and guidance from healthcare professionals, collectively shaped maternal decisions, consistent with the ecological model of breastfeeding behavior [21].

Overall, the findings underscore that exclusive breastfeeding practices in collectivist Muslim contexts cannot be fully understood through biomedical or individualistic frameworks alone. Instead, they require a more integrative perspective that considers the dynamic interplay of breastfeeding knowledge, emotional experiences, social support, cultural norms, and religious values. In line with this perspective, this study highlights that exclusive breastfeeding is a culturally embedded, socially negotiated process, shaped by knowledge, relational support, emotional regulation, and moral attitudes [24]. Interventions aiming to enhance breastfeeding should go beyond individual-focused education, incorporating family, community, and culturally sensitive support systems to align with existing social structures and improve sustainability of practices in collectivist contexts. Future research could explore scalable community-based strategies that integrate family and religious networks, as well as examine how maternal adaptive strategies evolve across diverse socio-cultural settings.

### 4.6. This Study Contributes a Culturally Grounded Integrative Framework for Understanding Exclusive Breastfeeding Among Banjar Mothers

This study offers several important novelties in the field of exclusive breastfeeding research by reframing breastfeeding not merely as a health behavior, but as a culturally embedded maternal experience. The findings demonstrate that exclusive breastfeeding is constructed as a relational and moral practice deeply rooted in family values, religious beliefs, and social norms within the Banjar community. Breastfeeding is interpreted not only as a biological function but also as an expression of maternal responsibility, emotional attachment, and moral commitment shaped by collectivist cultural structures. In this context, social support emerges as a complex and ambivalent mechanism. While it provides emotional reassurance and instrumental assistance, it simultaneously functions as a form of social regulation that may influence maternal autonomy in decision-making. This dual character of support expands existing perspectives by emphasizing that in collectivist societies, support is not always neutral, but embedded within normative expectations and relational obligations that shape maternal behavior.

Furthermore, this study advances the conceptualization of postpartum stress by demonstrating that it is not solely an intrapersonal psychological experience, but a relationally negotiated process shaped through interactions with spouses, extended family members, and healthcare professionals. This shifts the understanding of postpartum stress from an individual-centered framework toward a socially embedded phenomenon, where emotional regulation is co-constructed within relational networks. Building on these insights, the study proposes a culturally grounded integrative model illustrating how breastfeeding knowledge, cultural values, social support, postpartum stress, and maternal attitudes interact dynamically in shaping breastfeeding practices during the early postpartum period. This model provides a significant theoretical contribution by integrating cultural, relational, and psychological dimensions that are often treated separately in existing breastfeeding frameworks. From a practical perspective, the findings highlight the importance of designing culturally sensitive interventions that involve not only mothers but also family members and community actors, thereby enhancing the effectiveness and continuation of breastfeeding practices during the early postpartum period in collectivist settings.

Despite these contributions, this study has several limitations that should be acknowledged. First, the study is based on a relatively small and purposively selected sample of Banjar mothers, which may limit the generalizability of the findings. Second, the focus on the early postpartum period (0–6 weeks) captures initial breastfeeding experiences but may not reflect longer-term practices and challenges. Third, the study is situated within a specific cultural and regional context, which may limit the transferability of the findings to other populations. Additionally, potential sources of bias, including recruitment through healthcare facilities and the possibility of social desirability in participants’ responses, should be considered when interpreting the findings. Nevertheless, the study provides valuable context-sensitive insights into the socio-cultural dynamics shaping exclusive breastfeeding practices. Future research is encouraged to include larger and more diverse samples across multiple cultural settings to further explore postpartum breastfeeding experiences and validate these findings.

## 5. Conclusions

This study suggests that exclusive breastfeeding during the early postpartum period among Banjar mothers is not merely a health practice limited to infant nutrition, but rather a culturally embedded maternal experience shaped by social, emotional, and cultural meanings. Breastfeeding is constructed as a moral and religious responsibility, reflecting maternal identity and commitment within family and community contexts. The findings highlight that breastfeeding practices are shaped by a complex interplay of postpartum stress, social support, cultural beliefs, and normative expectations, which may simultaneously support and challenge mothers in sustaining exclusive breastfeeding. From both theoretical and practical perspectives, these findings underscore the importance of incorporating cultural, relational, and emotional dimensions into breastfeeding interventions. Efforts to promote exclusive breastfeeding should move beyond individual-focused approaches by integrating family, community, and culturally sensitive healthcare support. Overall, this study contributes to a more comprehensive and context-sensitive understanding of exclusive breastfeeding in collectivist Muslim settings, offering important insights for the development of maternal health policies and interventions. While acknowledging its contextual and methodological limitations, this study provides a meaningful basis for future research and practice in similar socio-cultural contexts.

## Figures and Tables

**Table 1:** Socio-cultural characteristics of the study setting.

Aspect	Description
Country Context	Indonesia as the fourth most populous country with high cultural, ethnic, and religious diversity; approximately 86% of the population is Muslim [40]
Social Structure	Predominantly collectivist society where individuals align with group norms and prioritize family and community interests [27,41]
Identity Formation	Individual identity shaped by social roles within family and community systems, including motherhood [28]
Study Location	South Kalimantan Province as a region with a strong cultural and ethnic identity
Ethnic Context	The Banjar ethnic group as the dominant population with strong kinship structures and hierarchical social relations [35]
Religious Context	High influence of Islamic values in shaping daily practices, including maternal roles and infant care
Cultural Meaning of Breastfeeding	Breastfeeding interpreted as both a health practice and a moral–religious obligation within Islamic and communal norms
Decision-Making Structure	Maternal decisions influenced by extended family members such as husbands, parents, and in-laws within a collectivist framework
Healthcare Context	Availability of maternal and child health services, including primary healthcare centers, alongside ongoing national breastfeeding promotion programs
Socio-Cultural Influence	Local cultural norms continue to shape perceptions, attitudes, and practices of breastfeeding, particularly during the early postpartum period (0–6 weeks)

**Table 2:** Participant selection criteria.

Category	Criteria
Inclusion Criteria	(1) Breastfeeding mothers of Banjar ethnicity residing in South Kalimantan; (2) aged 18–35 years; (3) mothers with infants aged 0–6 weeks postpartum (including both primiparous and multiparous mothers) (4) Muslim; (5) currently breastfeeding or intending to breastfeed; (6) willing to participate and provide informed consent
Exclusion Criteria	(1) Participants who did not complete or withdrew from the data collection process; (2) mothers or infants diagnosed with significant postpartum clinical complications that could substantially affect breastfeeding experiences
Sampling Technique	Purposive sampling based on relevance to research objectives
Recruitment Process	Identification through primary healthcare centers and community health posts (Puskesmas and Posyandu), followed by direct contact and invitation by the researcher
Final Sample Size	15 participants

**Table 3:** Participant characteristics.

Participant Code	Age (Years)	Parity Status	Infant Age (Weeks)	Mode of Delivery	Type of Birth	Education Level	Occupation	Household Structure	Breastfeeding Status	Formula Introduced
Mother 1	28	Multiparous	4	Vaginal	Singleton	Senior High School	Housewife	Nuclear Family	Breastfeeding	No
Mother 2	34	Primiparous	4	Cesarean	Singleton	Master’s Degree	Lecturer	Extended Family	Breastfeeding	No
Mother 3	35	Multiparous	5	Vaginal	Singleton	Diploma	Housewife	Extended Family	Breastfeeding	No
Mother 4	28	Multiparous	6	Vaginal	Singleton	Bachelor’s Degree	Therapist	Extended Family	Breastfeeding	Yes
Mother 5	33	Multiparous	5	Vaginal	Singleton	Diploma	Midwife	Extended Family	Breastfeeding	No
Mother 6	31	Multiparous	5	Cesarean	Singleton	Bachelor’s Degree	Teacher	Extended Family	Breastfeeding	Yes
Mother 7	27	Multiparous	5	Cesarean	Singleton	Bachelor’s Degree	Private Employee	Nuclear Family	Breastfeeding	Yes
Mother 8	29	Multiparous	3	Vaginal	Singleton	Senior High School	Housewife	Nuclear Family	Breastfeeding	No
Mother 9	31	Primiparous	2	Vaginal	Singleton	Diploma	Nurse	Nuclear Family	Breastfeeding	Yes
Mother 10	30	Multiparous	3	Vaginal	Singleton	Bachelor’s Degree	Private Employee	Nuclear Family	Breastfeeding	No
Mother 11	32	Primiparous	5	Cesarean	Singleton	Senior High School	Private Employee	Extended Family	Breastfeeding	Yes
Mother 12	30	Multiparous	2	Vaginal	Singleton	Junior High School	Housewife	Nuclear Family	Breastfeeding	No
Mother 13	34	Multiparous	4	Vaginal	Singleton	Diploma	Midwife	Nuclear Family	Breastfeeding	No
Mother 14	33	Primiparous	5	Vaginal	Singleton	Senior High School	Private Employee	Nuclear Family	Breastfeeding	No
Mother 15	30	Primiparous	5	Vaginal	Singleton	Senior High School	Housewife	Extended Family	Breastfeeding	Yes

**Table 4:** Summary of themes and representative quotations.

Theme	Key Meaning	Representative Quotation
Theme 1: Breastfeeding Knowledge	Basic understanding of breastfeeding, perceived benefits, and psychological concerns, including doubts about milk sufficiency	“Breastfeeding is a natural process between mother and baby and builds closeness.” (Mother 1)
Theme 2: Social Support	Emotional, informational, and practical support from husbands, family members, and healthcare providers	“My husband helps take care of the baby when I pump milk.” (Mother 3)
Theme 3: Postpartum Stress	Emotional distress characterized by confusion, guilt, anxiety, and formula-feeding dilemmas in the early postpartum period	“I was confused when my milk had not come out yet.” (Mother 13)
Theme 4: Attitudes toward Breastfeeding	Breastfeeding as a personal commitment shaped by values, beliefs, and rational–emotional considerations	“Breastfeeding is a commitment I made since pregnancy.” (Mother 1)
Theme 5: Exclusive Breastfeeding Practices	Adaptive strategies and environmental support that help mothers sustain exclusive breastfeeding practices	“Support from family makes me continue exclusive breastfeeding.” (Mother 1)

## Data Availability

The data presented in this study are available on request from the corresponding author. The data are not publicly available due to privacy and ethical restrictions.

## References

[1] World Health Organization, UNICEF (2014). Global Nutrition Targets 2025: Breastfeeding Policy Brief.

[2] Victora C.G., Requejo J.H., Barros A.J., Berman P., Bhutta Z., Boerma T., Chopra M., De Francisco A., Daelmans B., Hazel E. (2016). Countdown to 2015: A decade of tracking progress for maternal, newborn, and child survival. Lancet.

[3] World Health Organization (2012). Country Cooperation Strategy 2023–2027: Indonesia Continuity and Change. https://iris.who.int/server/api/core/bitstreams/c680e6cc-42a8-4fc6-9891-b2dae40bd7d3/content.

[4] UNICEF, World Health Organization (2023). Global Breastfeeding Scorecard 2023: Rates of Breastfeeding Increase Around the World Through Improved Protection and Support.

[5] Rollins N.C., Bhandari N., Hajeebhoy N., Horton S., Lutter C.K., Martines J.C., Piwoz E.G., Richter L.M., Victora C.G. (2016). Why invest, and what it will take to improve breastfeeding practices?. Lancet.

[6] Corrêa A.M.S., Uribe M.C.Á., Quiñonez H.M., Escamilla R.P. (2012). Escala Latinoamericana y Caribeña de Seguridad Alimentaria (ELCSA): Manual de Uso y Aplicaciones.

[7] Schmied V., Lupton D. (2001). Blurring the boundaries: Breastfeeding and maternal subjectivity. Sociol. Health Illn..

[8] Palmér L., Carlsson G., Brunt D., Nyström M. (2015). Existential security is a necessary condition for continued breastfeeding despite severe initial difficulties: A lifeworld hermeneutical study. Int. Breastfeed. J..

[9] Mercer R.T. (2004). Becoming a Mother Versus Maternal Role Attainment. J. Nurs. Scholarsh..

[10] Shorey S., Chee C.Y.I., Ng E.D., Chan Y.H., Tam W.W.S., Chong Y.S. (2018). Prevalence and incidence of postpartum depression among healthy mothers: A systematic review and meta-analysis. J. Psychiatr. Res..

[11] Falah-Hassani K., Shiri R., Dennis C.-L. (2017). The prevalence of antenatal and postnatal co-morbid anxiety and depression: A meta-analysis. Psychol. Med..

[12] Figueiredo B., Canário C., Field T. (2014). Breastfeeding is negatively affected by prenatal depression and reduces postpartum depression. Psychol. Med..

[13] Fallon V., Komninou S., Bennett K.M., Halford J.C.G., Harrold J.A. (2017). The emotional and practical experiences of formula-feeding mothers. Matern. Child Nutr..

[14] Brown A., Davies R. (2014). Fathers’ experiences of supporting breastfeeding: Challenges for breastfeeding promotion and education. Matern. Child Nutr..

[15] Spencer B.S., Grassley J.S. (2013). African American Women and Breastfeeding: An Integrative Literature Review. Health Care Women Int..

[16] Yunita P. (2024). Survey on Father’s: Knowledge About Breastfeeding. J. Heal. Res. Technol..

[17] Kelly P.J., Sidhu A., Sajja A., Majeethia D., Dodge E., Aboul-Enein B.H. (2025). Breastfeeding interventions and programs conducted in the Islamic Republic of Iran: A scoping review. Health Educ. Res..

[18] Balogun O.O., Dagvadorj A., Yourkavitch J., da Silva Lopes K., Suto M., Takemoto Y., Mori R., Rayco-Solon P., Ota E. (2017). Health Facility Staff Training for Improving Breastfeeding Outcome: A Systematic Review for Step 2 of the Baby-Friendly Hospital Initiative. Breastfeed. Med..

[19] Thulier D., Mercer J. (2009). Variables Associated with Breastfeeding Duration. J. Obstet. Gynecol. Neonatal Nurs..

[20] Borra C., Iacovou M., Sevilla A. (2015). New Evidence on Breastfeeding and Postpartum Depression: The Importance of Understanding Women’s Intentions. Matern. Child Health J..

[21] McFadden A., Gavine A., Renfrew M.J., Wade A., Buchanan P., Taylor J.L., Veitch E., Rennie A.M., Crowther S.A., Neiman S. (2017). Support for healthy breastfeeding mothers with healthy term babies. Cochrane Database Syst. Rev..

[22] Dykes F. (2005). ‘Supply’ and ‘demand’: Breastfeeding as labour. Soc. Sci. Med..

[23] Thomson G., Ebisch-Burton K., Flacking R. (2015). Shame if you do—shame if you don’t: Women’s experiences of infant feeding. Matern. Child Nutr..

[24] Singelis T.M., Triandis H.C., Bhawuk D.P.S., Gelfand M.J. (1995). Horizontal and Vertical Dimensions of Individualism and Collectivism: A Theoretical and Measurement Refinement. Cross Cult. Res..

[25] Hofstede G. (2001). Culture’s Recent Consequences: Using Dimension Scores in Theory and Research. Int. J. Cross Cult. Manag..

[26] Markus H.R., Kitayama S. (1991). Cultural Variation in the Self-Concept. The Self: Interdisciplinary Approaches.

[27] Groleau D., Soulière M., Kirmayer L.J. (2006). Breastfeeding and the cultural configuration of social space among Vietnamese immigrant woman. Health Place.

[28] Alianmoghaddam N., Phibbs S., Benn C. (2018). Reasons for Stopping Exclusive Breastfeeding Between Three and Six Months: A Qualitative Study. J. Pediatr. Nurs..

[29] Abdulrahman A., Osamah A., Abdulrahman A., Ahmed A.M. (2018). The Relation between Breastfeeding and Incidence of Diabetes Mellitus Type I in Saudi Arabia, Cross Sectional Study. Egypt. J. Hosp. Med..

[30] Liamputtong P. (2011). Focus Group Methodology: Principles and Practice.

[31] Taylor J.S., Kacmar J.E., Nothnagle M., Lawrence R.A. (2005). A Systematic Review of the Literature Associating Breastfeeding with Type 2 Diabetes and Gestational Diabetes. J. Am. Coll. Nutr..

[32] Geertz C. (1960). The Javanese Kijaji: The Changing Role of a Cultural Broker. Comp. Stud. Soc. Hist..

[33] Daud S., Yusoff W.F.W. (2011). How intellectual capital mediates the relationship between knowledge management processes and organizational performance?. Afr. J. Bus. Manag..

[34] Abbas E.W., Handy M.R.N., Shaleh R.M., Hadi N.T.F.W. (2020). Ecotourism of Martapura River Banjarmasin as a Learning Resources on Social Studies. Innov. Soc. Stud. J..

[35] Rahmawati C.E. (2024). Hubungan Pemberian ASI dan Pola Makan dengan Kejadian Stunting Pada Balita. J. Ilm. Bidan.

[36] Fitri S.H., Suara M. (2022). Pengaruh Terapi Aktivitas Kelompok (TAK) Stimulasi Persepsi Halusinasi Terhadap Tingkat Kecemasan pada Pasien Halusinasi Pendengaran di RS Jiwa Islam Klender Tahun 2022. J. Pendidik. Konseling.

[37] Creswell J.W., Poth C.N. (2016). Qualitative Inquiry and Research Design: Choosing Among Five Approaches.

[38] Ali M., Wiranto E.B., Shobahiya M., Maksum M.N.R. (2024). Culture, religion, and harmony: The struggle for roles in diversity in Indonesia. Rev. Gestão Soc. Ambient..

[39] Pelham B., Hardin C., Murray D., Shimizu M., Vandello J. (2022). A truly global, non-WEIRD examination of collectivism: The Global Collectivism Index (GCI). Curr. Res. Ecol. Soc. Psychol..

[40] Thornberg R., Perhamus L.M., Charmaz K. (2014). Grounded Theory.

[41] Waring M., Arthur J., Waring M., Coe R., Hedges L.V. (2012). Finding Your Theoretical Position. Research Methods and Methodologies in Education.

[42] Sugiyono (2019). Metode Penelitian Kuantitatif, Kualitatif R&D.

[43] Braun V., Clarke V. (2006). Using thematic analysis in psychology. Qual. Res. Psychol..

[44] Lincoln Y.S., Guba E.G. (1985). Naturalistic Inquiry.

[45] Pérez-Escamilla R., Curry L., Minhas D., Taylor L., Bradley E. (2012). Scaling Up of Breastfeeding Promotion Programs in Low- and Middle-Income Countries: The ‘Breastfeeding Gear’ Model. Adv. Nutr..

[46] Apriati Y., Alfisyah, Azkia L., Raudah (2020). Revitalisasi Folk Song (Nyanyian Rakyat) sebagai Media Penanaman Nilai Dikalangan Masyarakat Banjar Kalimantan Selatan. Solidarity.

[47] Susilawati E., Sari L.A. (2024). Empowerment of Pregnant Women Through Education and Training on Breastfeeding Techniques for Breastfeeding Preparation and Exclusive Breastfeeding in Penyengat Olak Village, Muaro Jambi Regency. Mattawang J. Pengabdi. Masy..

[48] Hedberg I.C. (2013). Barriers to Breastfeeding in the WIC Population. MCN Am. J. Matern. Child Nurs..

[49] Redhead D., Power E.A. (2022). Social hierarchies and social networks in humans. Philos. Trans. R. Soc. B Biol. Sci..

[50] Figueiredo B., Dias C.C., Pinto T.M., Field T. (2017). Exclusive breastfeeding at three months and infant sleep-wake behaviors at two weeks, three and six months. Infant Behav. Dev..

